# DAF-12 Regulates a Connected Network of Genes to Ensure Robust Developmental Decisions

**DOI:** 10.1371/journal.pgen.1002179

**Published:** 2011-07-21

**Authors:** Daniel Hochbaum, Yue Zhang, Carsten Stuckenholz, Paul Labhart, Vassili Alexiadis, René Martin, Hans-Joachim Knölker, Alfred L. Fisher

**Affiliations:** 1 Division of Geriatric Medicine, Department of Medicine, University of Pittsburgh, Pittsburgh, Pennsylvania, United States of America; 2 Department of Radiation Oncology, Beth Israel Deaconess Medical Center, Harvard Medical School, Boston, Massachusetts, United States of America; 3 Division of Hematology/Oncology, Department of Medicine, University of Pittsburgh, Pittsburgh, Pennsylvania, United States of America; 4 Active Motif, Carlsbad, California, United States of America; 5 Biocept, San Diego, California, United States of America; 6 ChiroBlock GmbH, Wolfen, Germany; 7 Department Chemie, Technische Universität Dresden, Dresden, Germany; Stanford University Medical Center, United States of America

## Abstract

The nuclear receptor DAF-12 has roles in normal development, the decision to pursue dauer development in unfavorable conditions, and the modulation of adult aging. Despite the biologic importance of DAF-12, target genes for this receptor are largely unknown. To identify DAF-12 targets, we performed chromatin immunoprecipitation followed by hybridization to whole-genome tiling arrays. We identified 1,175 genomic regions to be bound *in vivo* by DAF-12, and these regions are enriched in known DAF-12 binding motifs and act as DAF-12 response elements in transfected cells and in transgenic worms. The DAF-12 target genes near these binding sites include an extensive network of interconnected heterochronic and microRNA genes. We also identify the genes encoding components of the miRISC, which is required for the control of target genes by microRNA, as a target of DAF-12 regulation. During reproductive development, many of these target genes are misregulated in *daf-12(0)* mutants, but this only infrequently results in developmental phenotypes. In contrast, we and others have found that null *daf-12* mutations enhance the phenotypes of many miRISC and heterochronic target genes. We also find that environmental fluctuations significantly strengthen the weak heterochronic phenotypes of null *daf-12* alleles. During diapause, DAF-12 represses the expression of many heterochronic and miRISC target genes, and prior work has demonstrated that dauer formation can suppress the heterochronic phenotypes of many of these target genes in post-dauer development. Together these data are consistent with *daf-12* acting to ensure developmental robustness by committing the animal to adult or dauer developmental programs despite variable internal or external conditions.

## Author Summary

In *Caenorhabditis elegans* many life history decisions, such as interrupting reproductive development to form a dauer and the rate of aging, are under the control of the DAF-12 nuclear receptor. Recent work has started to elucidate the endocrine regulation of DAF-12, but few target genes for this receptor have been identified. In this study, we use chromatin immunoprecipitation to identify *in vivo* target genes for DAF-12. Among the targets are multiple genes involved in developmental progression, which are termed heterochronic genes, and subunits of the miRISC, which is required for the control of target genes by microRNA. In *daf-12* null worms, both heterochronic and miRISC genes are mis-regulated, but development is usually normal. This led us to examine whether DAF-12 normally acts to reinforce commitment to dauer or adult development and prevent disruption by environmental or genetic fluctuations. Consistent with this, we find that *daf-12* mutants are hypersensitive to environmental fluctuations and mutations in individual target genes. These results suggest a novel role for *daf-12* as a phenotypic capacitor that ensures commitment to reproductive or dauer development despite internal and external variations.

## Introduction

From bacteria to humans, organisms sense environmental cues and reprogram life history by altering gene expression patterns. In the nematode *C. elegans*, the nuclear receptor DAF-12 alters life history in response to the environment. During development, when conditions are unfavorable, due to starvation, crowding or high temperature, *daf-12* promotes diapause and formation of dauer larvae [1]–[3]. In favorable conditions, the DAF-12 receptor is activated by a group of steroidal carboxylic acids, termed dafachronic acids (DA) [4]–[6], produced from cholesterol via a multi-step pathway involving the *daf-36* Rieske-like oxygenase and the *daf-9* cytochrome P450 enzyme [7]–[9]. The activation of DAF-12 by DA leads to bypass of dauer arrest and expression of later larval developmental and reproductive programs [10]. On the other hand, in unfavorable conditions, DA is not produced, and DAF-12 binds to the co-repressor DIN-1 promoting dauer programs [11]. DAF-12 is also required for the normal lifespan of worms [12]–[14] and for the increased longevity of germline ablated animals [7], [15], [16].

DAF-12 also alters life history by regulation of the heterochronic circuit. The heterochronic pathway in *C. elegans* determines specific cell fate programs for each larval stage [17]. Several *daf-12* alleles, especially those in phenotypic class 1, have heterochronic phenotypes [10]. These mutants repeat L2 programs instead of progressing to L3 programs, resulting in excessive hypodermal seam cell division in the L3 stage and defects in distal tip cell migration in the L4 stage [1], [10]. The heterochronic phenotypes depend on the activity of the co-repressor *din-1*, as *daf-12(rh61);din-1* mutants develop normally [11]. In part, DAF-12 regulates the heterochronic circuit by controlling expression of *let-7* family miRNAs [18], [19], which are necessary to promote L3 and adult programs through the down-regulation of the heterochronic genes *hbl-1* and *lin-41* respectively [20], [21]. DAF-12 might also affect the heterochronic circuit via dauer formation, as many heterochronic phenotypes are suppressed in post dauer development [22]–[25].

Previously, Shostak *et al* identified DAF-12 binding sites *in vitro*. However, a small number of genes were described and the *in vivo* relevance of these target genes was not always clear. To identify novel genes regulated by DAF-12, we developed transgenic animals expressing an epitope-tagged *daf-12*-transgene. These worms were used to perform chromatin immunoprecipitation followed by hybridization of the precipitated DNA to whole genome tiling arrays. From the arrays, we identified 1175 genomic regions bound to DAF-12 and these regions are within 5 Kb of 3179 genes. Importantly, we detected binding sites in multiple heterochronic genes, miRISC (miRNA-induced silencing complex) genes, and a total of 40 miRNAs, suggesting that DAF-12 controls developmental progression through the direct regulation of these targets.

Biological systems are subject to mutations and environmental variation during development, but still are able to ensure a stereotyped developmental process. This is possible because organisms can buffer many perturbations and produce an invariant output. This capacity is called ‘robustness’ [26]. Since many heterochronic and miRISC genes found in our array were mis-regulated in a *daf-12(0)* null allele *daf-12(rh61rh411)*, but did not result in abnormal development [10], we hypothesized that DAF-12 could have a role in developmental robustness. Consistent with this idea, we found that *daf-12(0)* worms can enhance multiple heterochronic phenotypes, indicating that *daf-12* null worms are sensitized to genetic mutations. Further, we found that *daf-12(0)* worms are sensitized to environmental variability and show stronger heterochronic phenotypes in variable instead of constant conditions. In addition, multiple DAF-12 target genes were repressed in dauer, suggesting that in diapause DAF-12 can ‘reset’ the heterochronic circuit to ensure a stereotyped developmental program. These results illustrate a role for DAF-12 in the commitment to reproductive versus dauer development and suggest a novel function for this nuclear receptor to ensure a stereotyped phenotype despite internal and external variability.

## Results

### Use of a *daf-12* transgenic worm for chromatin immunoprecipitation

**Figure 1:**
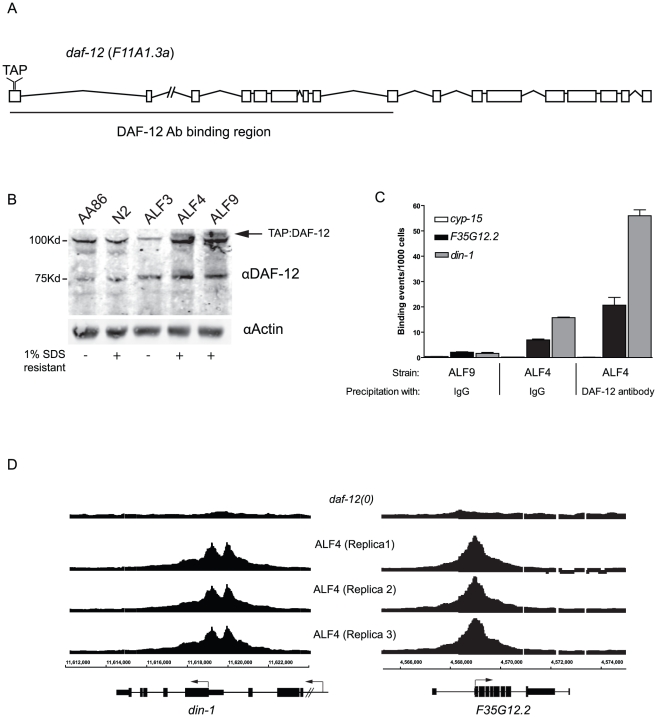
Generation of DAF-12 transgenic worms. (*A*) Schematic of *daf-12* transcript showing the TAP-tag and region recognized by the anti-DAF-12 antibody in the ligand binding domain (LBD). (*B*) Expression of the transgene in ALF4 and ALF9 lines was confirmed by Western blot using an anti-DAF-12 antibody. Actin was used as a loading control. The function of the TAP-tagged *daf-12* transgene was confirmed by incubation of starved worms with 1% SDS. (*C*) TAP-tagged DAF-12 bound chromatin was precipitated from ALF9 and ALF4 with an anti-DAF-12 antibody or IgG sepharose. Regions of interest were quantified by qPCR. (*D*) Binding sites within *din-1* and *F35G12.2* were visualized in tiling arrays using DNA from three replicas (ALF4) and control (*daf-12(0)*) with the Integrated Genome Browser software (Affymetrix). Arrows indicate initiation codons.

To identify target genes for DAF-12, we developed transgenic animals expressing a TAP-tagged transgene in a *daf-12(rh61rh411)* (*daf-12(0)*) background (Figure 1A). The tandem affinity purification, or TAP-tag, was previously used successfully to identify nicotinic receptor-associated proteins in *C. elegans* [27], [28]. The TAP-tag was inserted in-frame in *daf-12* exon 1 via homologous recombination [29] as insertion of a GFP-tag at this site produced a functional GFP:DAF-12 fusion protein [10]. By biolistic bombardment of ALF3 (*daf-12(rh61rh411)*; *unc-119(ed3)*), we obtained two integrated lines, ALF4 and ALF9. In each of these strains, DAF-12 is produced solely from the transgene. During the generation of our transgenic lines, a new commercially available DAF-12 antibody was released and this antibody was able to detect the fusion protein (Figure 1B). However, likely due to low DAF-12 expression in N2, endogenous DAF-12 was not detected. This suggests that the *daf-12* transgene is over-expressed relative to the endogenous levels. The transgene also rescues the *daf-12(0)* null allele, as starved transgenic worms form SDS resistant dauers (Figure 1B), suggesting that both transgenic lines express a functional *daf-12* transgene.

We performed pilot ChIP-chip experiments with ALF9 using the TAP tag and precipitation with IgG sepharose. In two replicas, we identified 52 genes as potential DAF-12 targets (data not shown). To identify additional target genes, we then optimized the assay by comparing ALF9 to ALF4 using IgG sepharose that recognized the TAP tag or the anti-DAF-12 antibody. Regions of no binding (*cyp-15*) and high affinity binding (*F35G12.2* or the DAF-12 co-repressor *din-1*) were quantified in both ALF9 and ALF4. Both the TAP and DAF-12 antibody precipitate the same regions in two separate transgenic strains (Figure 1C). Importantly, the strongest binding to *F35G12.2* and *din-1* was obtained using ALF4 and the DAF-12 specific antibody. Based on these findings, this combination was used to perform the ChIP-chip studies. We did not use the DAF-12 antibody for ChIP-chip studies with N2 worms, as these seemed less likely to be successful due to the very low level of endogenous DAF-12 expression.

To identify binding regions, arrays from three independent worm collections were analyzed and compared with *daf-12(0)*. We chose L2 and adult stages because *daf-12* is known to perform important and separate functions at these stages. Its activity at L2 is important for the dauer decision and developmental progression, whereas in the adult it is known to be required for influencing longevity [30]. Chromatin from ALF4 was collected at middle L2 and adult stages and mixed together for further analysis. As shown in Figure 1D, DAF-12 binding to *din-1* and *F35G12.2* is highly reproducible, as each of the replicas shows similar binding regions and intensity (ChIP enrichment ratio of 16 and 21 respectively).

### Global analysis of DAF-12 binding sites

From the resulting tiling array data, peaks representing DAF-12 binding sites were identified using TAS software (Affymetrix) (see Materials and Methods for a complete description). The data reported in this publication have been deposited in the NCBI's Gene Expression Omnibus accession number GSE28350 (http://www.ncbi.nlm.nih.gov/geo/query/acc.cgi?acc=GSE28350) [31]. To be considered a legitimate DAF-12 target, we required the peak to be present in all three replicates and not in a *daf-12(0)* negative control. This resulted in 1175 genomic regions bound to DAF-12. 1155 were within 5 Kb of 3179 genes and just 20 genomic regions (1.7%) were not associated with any known gene (Table S1). We also repeated the analysis looking for genes within 1 Kb and within 2 Kb of the binding sites (Table S1). Also, a table for binding regions when triplicates were positives and *daf-12(0)* was also positive is found in Table S2. The number of candidate target genes is lower but comparable in magnitude to the 6018 target genes recently identified for PHA-4, another developmental regulator in *C. elegans*, via chromatin immunoprecipitation followed by sequencing (ChIP-seq) [32]. Furthermore, approximately 20% of previously identified DAF-12 targets by Shostak *et al* [33] were also found in our ChIP-chip analysis (not shown), including the *lit-1* gene, which was extensively validated in their study.

**Figure 2:**
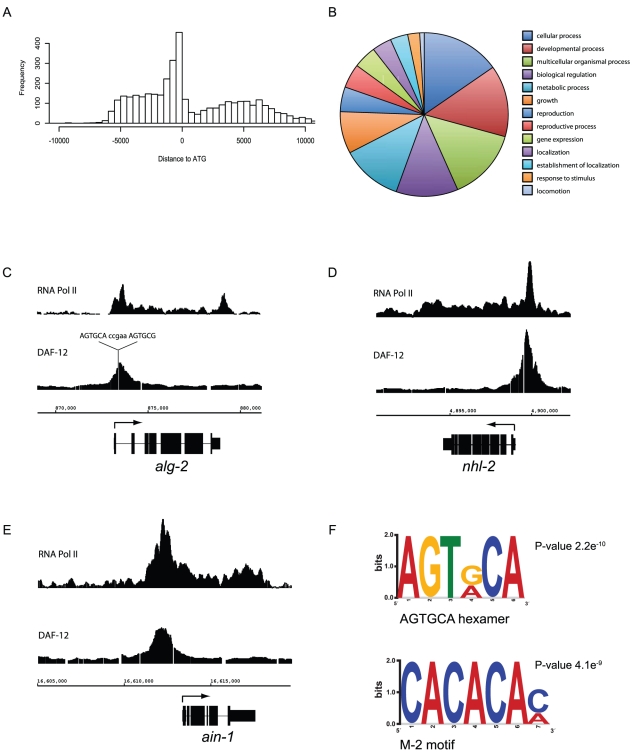
Analysis of DAF-12 binding sites. (*A*) Distribution of the distance between DAF-12 binding sites and candidate gene targets (ATG). (*B*) Pie chart illustrates gene ontology for candidate target genes defined by biological function. (*C*) RNA pol II and DAF-12 binding sites in L2 stage animals were compared within the miRISC gene *alg-2* using IGB software. A DR5 sequence within the DAF-12 binding peak is shown. (*D*) Analysis of the miRISC gene *nhl-2*. A total of 15 AGTGCA-related hexameric sequences were found. (*E*) Analysis of the miRISC gene *ain-1*. Multiple M-2 motifs were found. (*F*) AGTGCA-related sequences and M-2 motifs were over-represented among DAF-12 binding regions as identified by the MEME and DREME programs. The statistical significance of the motifs determined by p-values is also shown.

These DAF-12 binding sites tended to cluster in proximal upstream regions with a smaller number inside or downstream of genes (Figure 2A). The identified DAF-12 target genes are involved in diverse biological functions based on gene ontology associations, including development, metabolism, growth and gene expression (Figure 2B). Examples of these targets include heterochronic genes (Representation Factor = 6.9 *p*<1.64×10^−10^), all identified components of the miRISC complex (RF = 9.4 *p*<1.46×10^−6^), including regulatory factors such as *nhl-2* and *xrn-2* [34], [35], genes required for autophagy (RF = 7.1 *p*<3.014e^−04^) and a total of 51 ribosome subunits (*rpl* and *rps*) (RF = 1.7 *p*<2.268e^−04^). Genes involved in development, ribosomal function, mitochondrial function, and aging were found to be particularly over-represented (Table S3) via analysis of the targets with the DAVID program [36].

To examine whether DAF-12 binding sites are gene regulatory regions, we compared DAF-12 binding sites in the miRISC genes *alg-2*, *nhl-2* and *ain-1*, with RNA Pol II binding sites from L2 stage larva as a proof of principle [32]. As expected, DAF-12 binding is localized to upstream promoter regions and associated with the recruitment of RNA Pol II binding, suggesting that DAF-12 is associated with regions of active transcription (Figure 2C–2E). The peak within the *alg-2* promoter contains the DR5 repeat, sequence 5′-AGTGCAccgaaAGTGCG-3′, a very high affinity DAF-12 binding site [33] comprised of two hexameric DAF-12 binding sites separated by 5 bp (Figure 2C). The *nhl-2* promoter does not contain DR repeats but a total of 15 hexameric sequences were found (Figure 2D). The *ain-1* promoter contains just a single hexameric sequence, but has multiple M-2 motifs (CACACA), a previously identified DAF-12 binding site (Figure 2E) [37]. When analyzed globally, approximately 30% of DAF-12 target genes are actively transcribed in the L2 stage (not shown), which may reflect target genes being active at stages other than L2 or transcriptional repression instead of activation of a subset of target genes.

We then analyzed DAF-12 binding regions to find *de novo* motifs by MEME and DREME, which gives a minimal MEME-formatted motif [38]. Known DAF-12 binding sites, such as the hexameric sequences identified by Shostak *et al* [33] and the M-2 motif identified by Ao *et al* [37] were over-represented (Figure 2F). Together these results confirm that the binding sites found in our array contain known DAF-12 binding sites, which is consistent with them being bound *in vivo* by DAF-12.

The consensus sequence GAGAGA (Figure S1) was also over-represented among binding regions. Interestingly, this GAGA element was also identified as being enriched in PHA-4 binding sites identified by ChIP-seq [32], [37]. DAF-12 and PHA-4 have been shown to co-regulate genes involved in pharynx development and we found that this interaction may be more widespread as they share a significant number of target genes (Figure S2).

### DAF-12–dependent regulation of target genes

**Figure 3:**
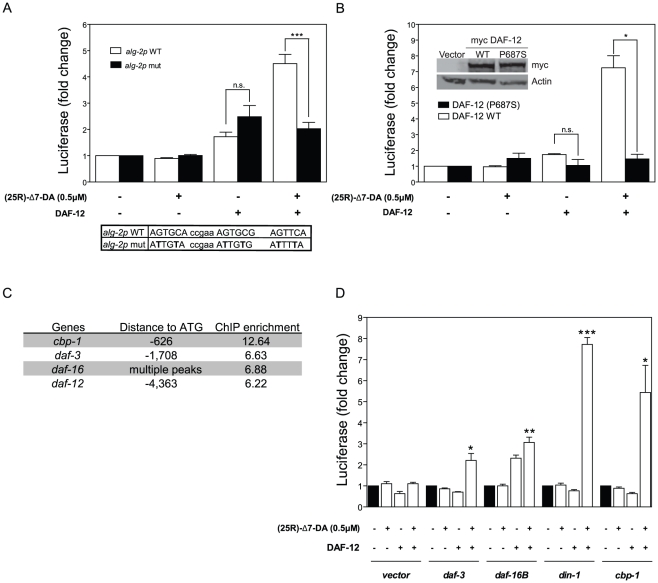
DAF-12 activates target genes in cells. (*A*) HEK cells were transfected with either pCMV-myc-DAF-12 or pCMV-myc vector, along with pGL3-Luc *alg-2* promoter and pRL-TK *renilla* to normalize for transfection efficiency. Cells were stimulated for 16 hr with 0.5 µM (25R)-Δ7–DA or DMSO as a control. Mutations within DR5 domain are indicated (box). (*B*) Luciferase activity was measured for pGL3-Luc *let-70/atg-7* promoter as in (A), with either DAF-12 or DAF-12 (P687S) expression plasmids. (*Inset*) Western blot shows similar expression levels of WT and mutant DAF-12 proteins. (*C*) Binding sites and ChIP enrichment for DAF-12 relative to ATG within genes involved in transcriptional regulation and dauer formation. (*D*) Promoter regions of corresponding genes were subcloned into pGL3-Luc and luciferase activity was measured as in (A). Bars denote standard error of mean (SEM). P-values were determined by Student's t-test. * *p*<0.05, ** *p*<0.01, *** *p*<0.001, *n.s.* (difference is not statistically significant).

To test whether binding sites in our array represent DAF-12 response elements, we transfected HEK cells with luciferase reporter constructs along with a myc-tagged DAF-12 expression plasmid [18]. This cell-based assay has the advantage of directly measuring agonist-dependent activation with DA, as opposed to functional assays in *S. cerevisiae* not involving agonist treatment [33] or worm transgenes, which could be regulated by transcription factors other than DAF-12. Among the targets we identified is the miRISC gene *alg-2* whose 2 Kb promoter region is sufficient for agonist-dependent activation (Figure 3A). The *alg-2* promoter contains a DR5 sequence that is required for activation (Figure 3A, box). Interestingly, both the WT promoter and the mutant *alg-2* promoter that lacks the DR5 domain exhibit weak DAF-12-dependent activation in the absence of DA. This transcriptional activation, which does not require the DR5 domain, could be due to DAF-12 tethering. This activation mechanism was observed for other nuclear receptors, such as ER or GR, where adaptor molecules, like SP1 or AP-1 can recruit the nuclear receptor to the DNA and induce transcriptional activation independent of the agonist [39]. Alternatively, over-expressed DAF-12 could bind to low affinity sites, such as incomplete M-2 motifs or GAGA motifs present in the promoter (Figure S1). Regardless, our findings demonstrate that these play a minor role compared to DAF-12 actions through the DR5 domain.

Another putative target is the promoter region of the CEOP4404 operon, which contains the *atg-7* and *let-70* genes. As shown in Figure 3B, a 2 Kb promoter region for *atg-7* and *let-70* is strongly regulated by DAF-12 in cells. To address if this activation is due to DAF-12 recruiting a co-activator, we introduced a point mutation (P687S) within the DAF-12 AF-2 domain, which for other nuclear receptors is involved in recruiting co-activators after ligand binding [40]. This mutation is present in the *daf-12*(*rh284*) allele and causes a weak Daf-c phenotype at 25°C, perhaps due to impaired DAF-12 transcriptional activity [10]. As seen in Figure 3B, DAF-12 (P687S) is unable to promote transcriptional activity, even though expression of both proteins is similar (Figure 3B, inset).

We then analyzed DAF-12 target genes previously reported to be involved in dauer formation (Figure 3C). 2 Kb promoter regions of these genes were subcloned upstream of a luciferase gene reporter and transfected in HEK cells. As seen in Figure 3D, all promoter regions show agonist dependent activation, including the DAF-16 CBP/p300 co-activator CBP-1 [41]. These results suggest that DAF-12 could regulate dauer formation in part by regulation of *daf-3*, *din-1* and *daf-16* expression, but also by regulation of *daf-16* activity through controlling CBP-1 expression. Furthermore, DAF-12 could also regulate *daf-16* activity by controlling the expression of *akt-1*, *akt-2* and *pdk-1* [42] (Table S1). When considered together, these results demonstrate that the regions identified by ChIP-chip respond *in vivo* to DAF-12.

### DAF-12 regulates post-embryonic development

**Figure 4:**
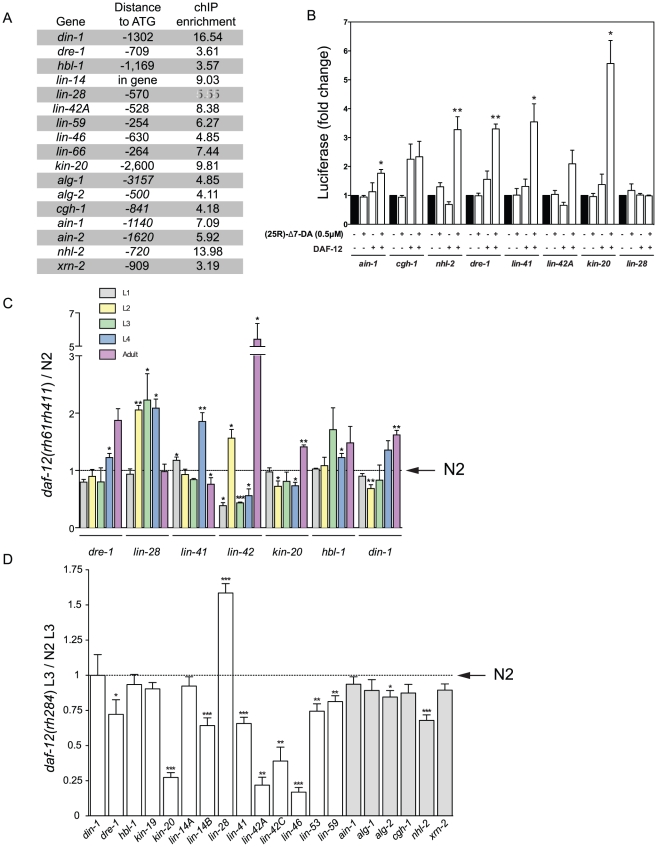
DAF-12 regulates heterochronic and miRISC gene expression. (*A*) Binding sites and ChIP enrichment for DAF-12 relative to ATG within heterochronic and miRISC genes. (*B*) Promoter regions of corresponding genes were subcloned into pGL3-Luc and luciferase activity was measured in HEK cells upon stimulation for 16 hr with 0.5 µM (25R)-Δ7–DA or DMSO as a control. (*C*) mRNA levels from synchronized *daf-12(rh61rh411)* or N2 worms were determined by RT-qPCR. Fold difference is shown relative to N2 mRNA levels (dotted line). (*D*) mRNA levels from synchronized *daf-12(rh284)* or N2 worms at L3 stage were determined by NanoString analysis for several heterochronic genes (white bars) or miRISC genes (grey bars). Fold difference is relative to N2 mRNA levels (dotted line). Bars denote standard error of mean (SEM). P-values were determined by Student's t-test. * *p*<0.05, ** *p*<0.01, *** *p*<0.001.

Proper regulation of the heterochronic circuit is essential for normal development [43]. Mutation in these heterochronic genes can cause either a precocious (skipping stage-specific events) or retarded (reiterating stage-specific events in later stages) developmental phenotype. In addition, miRISC components, required for mRNA degradation or translational repression are also essential for proper development as miRNAs regulate the heterochronic circuit [20]. As shown in Figure 4A, multiple heterochronic genes and all known miRISC genes contain DAF-12 binding sites, suggesting that DAF- 12 can directly regulate their expression.

Regulatory regions of multiple heterochronic genes, such as the F box gene *dre-1* [24], the RBCC gene *lin-41* [44], the RNA binding gene *lin-28* [45] and the circadian clock like genes *lin-42A* and *kin-20* (Figure S3) [46], [47] were subcloned upstream of a luciferase reporter. As shown in Figure 4B, all promoter regions tested, except the RNA binding gene *lin-28*, were activated by DAF-12 in an agonist-dependent fashion. The same analysis was performed for members of the miRISC complex. As shown in Figure 4B, the decapping gene *cgh-1* [48] was activated in a DAF-12-dependent agonist-independent fashion, representing perhaps another example of nuclear receptor tethering (Figure 3A). Also, the *argonaute* interacting protein, *ain-1*, shows a modest but reproducible activation (Figure 4B). In addition, *alg-2* seems to be a DAF-12 target (Figure 3A), as well as NHL-2, a protein that binds to ALG-1 and ALG-2 to modulate miRNA potency [35] (Figure 4B).

We then examined the *in vivo* effects of *daf-12* on heterochronic gene expression. Wild type (N2) and *daf-12(0)* worms were collected at different developmental stages, and heterochronic gene expression was quantified by RT-qPCR. As seen in Figure 4C, *daf-12(0)* worms show significant differences in expression when compared to N2. Importantly, DAF-12 can either repress or activate the same gene depending on the developmental stage (i.e. *lin-41* at L4 or adult stage). Interestingly, the circadian clock gene *lin-42*, which has been suggested to be important for the molting process [47], is normally repressed by DAF-12 at the adult stage. This could be necessary to prevent extra molting cycles once development is completed.

As many heterochronic genes were mis-regulated in L3, we focused on this stage. We compared mRNA levels of different heterochronic and miRISC genes from N2 or *daf-12*(*rh284*), a DAF-12 mutant unable to promote transcriptional activity (Figure 3B), using NanoString analysis [49]. As shown in Figure 4D, although all observed differences were not statistically significant, in most cases DAF-12 is necessary for full expression of these targets. However, DAF-12 might be involved in repression of *lin-28* as its expression is up-regulated in both *daf-12* mutants at the L3 stage (Figure 4C and 4D). Consistent with this, *lin-28* mRNA is also up-regulated in *din-1(dh149)* worms (data not shown). Our findings point to a role for DAF-12 in regulation of heterochronic and miRISC gene expression during post-embryonic development.

**Figure 5:**
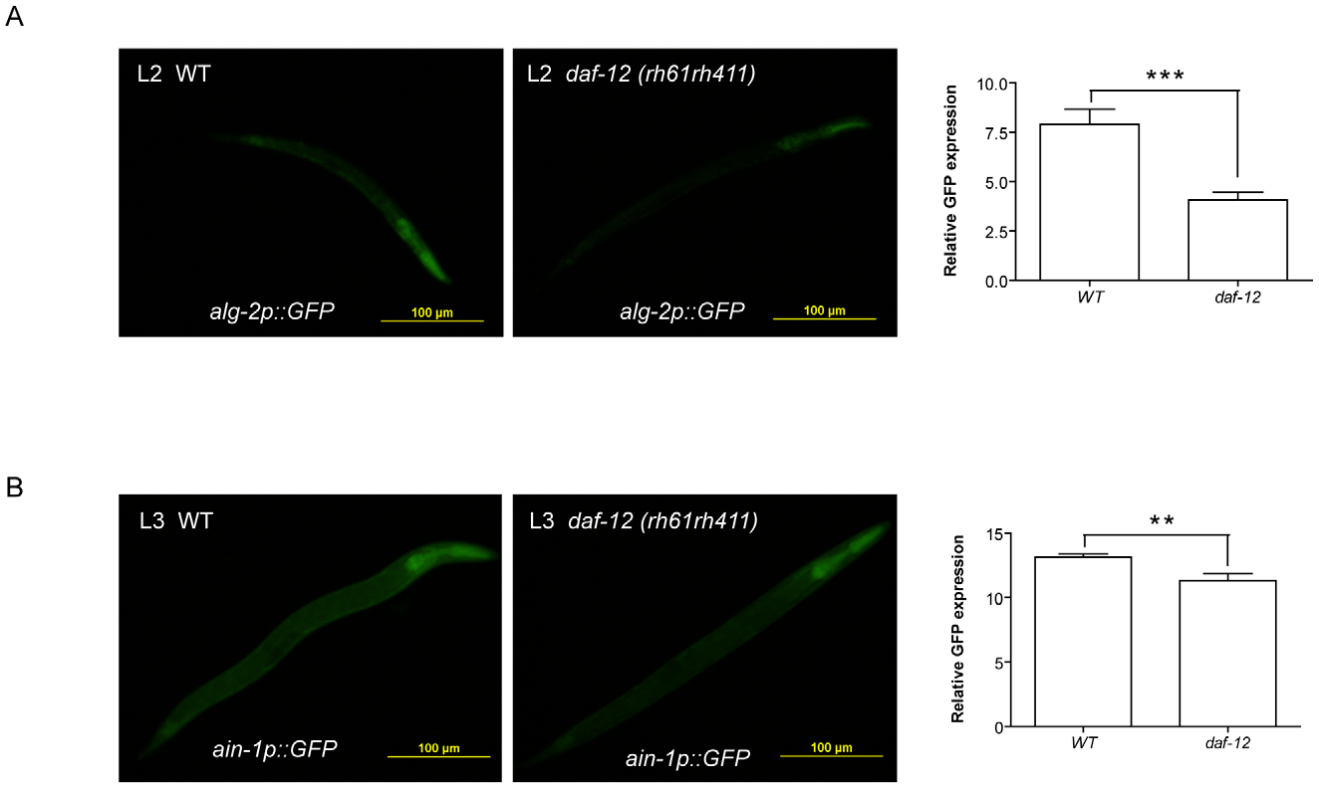
DAF-12 regulates expression of miRISC genes. (*A*) Image of *alg-2p::GFP* in WT or *daf-12(rh61rh411)* strains at the L2 stage (left). Graph showing *alg-2p::GFP* quantification in both strains (right) (*p*<0.0001). (*B*) Image of *ain-1p::GFP* in WT or *daf-12(rh61rh411)* strains at the L3 stage (left). Graph showing *ain-1p::GFP* quantification in both strains (right) (*p* = 0.002). P-values were determined by unpaired t-test.

Although all known miRISC genes contain DAF-12 binding sites, most of them showed a weak mis-regulation (Figure 4D). To test whether this occurs because *daf-12(rh284)* still retains some aspect of DAF-12 activity, or because the mis-regulation of these genes is tissue-specific, we created transgenic animals carrying *alg-2* and *ain-1* promoter regions upstream of GFP in WT or *daf-12(0)* mutants. As seen in Figure 5A, *daf-12(0)* worms at L2 stage show impaired *alg-2* expression most notably in the pharynx when compared to WT. Further, expression of *ain-1* at L3 stage was also impaired in *daf-12(0)* worms (Figure 5B). Taken together, these results suggest that DAF-12 controls the expression of miRISC components during reproductive development.

### *daf-12(0)* worms are sensitized to genetic mutations

**Figure 6:**
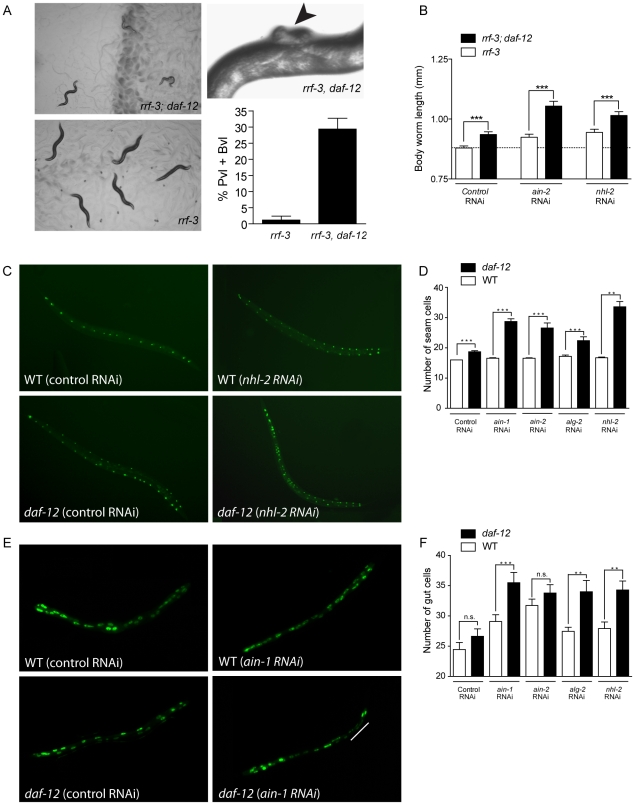
*daf-12(0)* worms are sensitized to changes in miRISC activity. (*A*) (Left panels) Images of *ain-1* RNAi treated *rrf-3* or *rrf-3; daf-12(rh61rh411)* worms. (Upper right) Photo showing protruding vulva phenotype (arrow). Graph showing effects of *ain-1* RNAi treatment on vulva development. (*B*) Body length was analyzed in *rrf-3* or *rrf-3; daf-12(rh61rh411)* worms treated with *ain-2* or *nhl-2* RNAi. (*C*) Effect of control and *nhl-2* RNAi on WT or *daf-12(rh61rh411)* worms carrying SCM::GFP transgene marking seam cells. (*D*) Quantification of seam cells in worms treated with miRISC RNAi. (*E*) Effect of *ain-1* RNAi on WT or *daf-12(rh61rh411)* worms carrying an *elt-2::GFP* transgene marking intestinal nuclei. White bar indicates abnormal extra division. (*F*) Quantification of intestinal cells in worms treated with miRISC RNAi. Bars denote standard error of mean (SEM). P-values were determined by Student's t-test. ** *p*<0.01, *** *p*<0.001, *n.s.* (difference is not statistically significant).

Although many genes involved in development were found in our array, in *daf-12(0)* mutants development usually occurs normally [10]. Based on this observation, we hypothesized that DAF-12 could have a role in developmental robustness. To address this hypothesis, we performed perturbation experiments [50] using the *rrf-3* mutant, which is hypersensitive to RNAi [51]. Both *rrf-3* and *rrf-3;daf-12* were exposed to miRISC gene RNAi, to see whether loss of *daf-12* could unmask developmental defects. When *rrf-3* worms were exposed to *ain-1* RNAi, only 3–4% developed protruding (Pvl) or burst vulva (Bvl) phenotypes. In contrast, approximately 30% of *rrf-3;daf-12* animals showed a penetrant vulva phenotype (Figure 6A). No vulva phenotype was observed for *rrf-3* or *rrf-3;daf-12* animals when exposed to control RNAi (not shown).

Treatment of *rrf-3* worms with *ain-1*, *ain-2*, *alg-2* or *nhl-2* RNAi produced an increase in body length (Figure 6B and data not shown). This result suggests that the known increased body length of *daf-12(0)* worms [13] could be due to the down-regulation of miRISC genes. However, when *daf-12(0)* worms were fed *nhl-2* or *ain-2* RNAi, a synergistic response was observed (Figure 6B).

During development, most hypodermal seam cells undergo a single asymmetric cell division. One daughter cell fuses to the hypodermal syncytium, while the other remains undifferentiated as a seam cell. However, in L2 stage the seam cells V1–4, V6 and H1 undergo a symmetric cell division before the asymmetric division, increasing the number of seam cells from 10 to 16 per side at the L/A switch [52]. Some *daf-12* class 1 alleles repeat L2 programs resulting in excessive hypodermal seam cells, but this phenotype is weaker in *daf-12(0)* animals [10]. As a result, we tested whether miRISC genes interacted with DAF-12 in the control of seam cell division. As shown in Figure 6C, while *nhl-2* knock-down induced a modest increase in seam cell number, a synergistic phenotype was revealed in *daf-12(0)*, as visualized with a nuclear localized SCM::GFP transgene. Furthermore, this synergistic response was also seen when other miRISC genes were down-regulated (Figure 6D).

*C. elegans* larvae hatch with 20 intestinal nuclei, and after 10–14 nuclear divisions in the L1 stage, reach 30–32 nuclei in the adult [53]. We studied postembryonic divisions in intestinal cells as visualized with a nuclear localized *elt-2::GFP* reporter. As shown in Figure 6E, *ain-1* knock-down induced a modest increase in number of intestinal nuclei cells. In contrast, *daf-12(0)* worms exhibited a synergistic phenotype upon treatment with *ain-1* RNAi (Figure 6E) or with RNAi for other miRISC genes (Figure 6F).

These results show that *daf-12(+)* worms can compensate for the loss of miRISC function to ensure normal development. However, *daf-12(0)* worms are unable to buffer these mutations. Further, previous observations have shown that *daf-12(0)* worms demonstrate enhanced developmental defects in response to mutations to multiple heterochronic genes that are also DAF-12 targets (Figure S4). Perhaps the combined mis-regulation of heterochronic genes and impaired miRNA activity, due to the control of miRNA [18] and miRISC expression by DAF-12, inhibits the ability of *daf-12(0)* worms to buffer genetic mutations during development. These findings point to a role for DAF-12 in ensuring developmental robustness during reproductive development, which we further test below.

### DAF-12 represses heterochronic and miRISC genes during dauer formation

**Figure 7:**
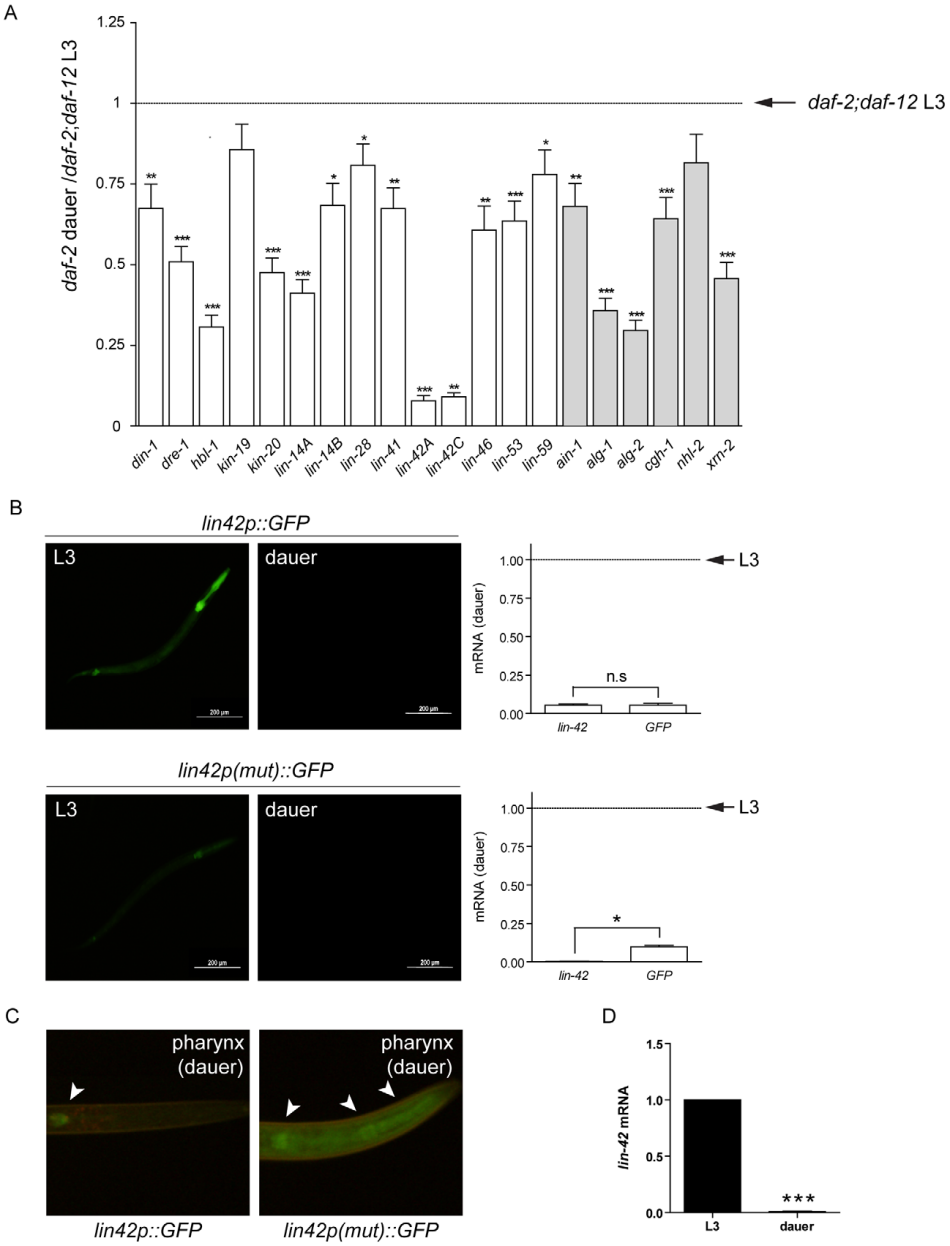
DAF-12 represses expression of target genes during dauer formation. (*A*) mRNA levels for heterochronic genes (white bars) or miRISC genes (grey bars) in L3 worms (*daf-2; daf-12(rh61rh411)*) or dauers (*daf-2*) grown at 25°C were quantified by NanoString analysis. The fold change for *daf-2* dauer relative to L3 levels are shown (*B*) Images of *lin-42p::GFP* or *lin-42p(mut)::GFP* worms in L3 stage (left panels) or dauer stage (right panels). Expression of *gfp* or endogenous *lin-42* in L3 and dauers stage worms as shown by RT-qPCR (graphs). (*C*) Detail of the pharynx from transgenic worms dauer arrested with increased exposure. Arrows indicate GFP expression. (*D*) Expression of endogenous *lin-42* in L3 and dauer stage worms as shown by RT-qPCR. Bars denote standard error of mean (SEM). P-values were determined by Student's t-test. * *p*<0.05, ** *p*<0.01, *** *p*<0.001.

The results presented above show that DAF-12 plays a key role in post-embryonic development and directly regulates the heterochronic circuit, miRISC genes and miRNA expression. We reasoned that these target genes might be repressed in diapause, as DAF-12 binds to the co-repressor DIN-1 to promote dauer formation [11]. To test if DAF-12 target genes are repressed in diapause, synchronized *daf-2(e1371)* or *daf-2(e1371);daf-12(rh61rh411)* worms were grown at 25°C. At this temperature, *daf-2* form dauers while *daf-2;daf-12* double mutants continue reproductive development. Worms at L3 stage or dauers were collected for mRNA quantification using NanoString analysis. As seen in Figure 7A, both miRISC and heterochronic genes are strongly repressed in dauer larvae relative to L3, suggesting that during reproductive development DAF-12 induces expression of these genes, whereas in dauer formation these genes are instead silenced.

To determine if DAF-12 is involved in repression of these genes, we created transgenic worms carrying a 2 Kb promoter region from *lin-42A* upstream of a GFP reporter. The same promoter region was subjected to mutagenesis to eliminate DAF-12 binding sites, such as DR and M-2 domains (Figure S5) [33], [37]. We have chosen to study *lin-42A* for multiple reasons. DAF-12 regulates *lin-42A* expression in every developmental stage (Figure 4C), *lin-42* is strongly repressed in diapause (Figure 7A) and down-regulation is necessary for dauer formation [54]. As shown in Figure 7B, in L3 stage both WT and mutant promoters drove GFP expression mainly in the pharynx and the tail, and GFP expression was occasionally observed in the intestine. GFP expression was strongly reduced in dauers when compared with the L3 stage for both reporter constructs (Figure 7B). Consistent with this, endogenous *lin-42* was repressed approximately 1000× when compared N2 worms at the L3 or dauer stages (Figure 7D). When endogenous *lin-42* and *lin-42p::GFP* were analyzed by RT-qPCR, GFP and *lin-42* transcripts were similarly repressed when they form dauers (Figure 7B, upper graph). However, when endogenous *lin-42 and* the *lin-42p(mut)::GFP* were compared, the *GFP* transcript retained 25-fold more expression than the endogenous *lin-42* transcript (Figure 7B, lower graph), suggesting that DAF-12 binding sites are strictly required for a full repression of *lin-42* in diapause. This differential GFP expression in dauers was observed at a higher exposure and magnification of the pharynx (Figure 7C).

These results show that DAF-12 represses the heterochronic circuit to commit to dauer formation. Interestingly, many heterochronic phenotypes, such as those due to *daf-12*, *dre-1*, *hbl-1*, *lin-4*, *lin-14*, *lin-28*, *lin-42*, and *lin-58* are suppressed after dauer formation [1], [22]–[25] (Figure S6). Together these results suggest that inhibition of the heterochronic circuit by DAF-12 could be necessary to interrupt development and enter diapause. In addition, this could be necessary to reset the heterochronic circuit to progress through development after worms exit dauer, adding another level of regulation to ensure developmental robustness.

### DAF-12 acts as a developmental capacitor

**Figure 8:**
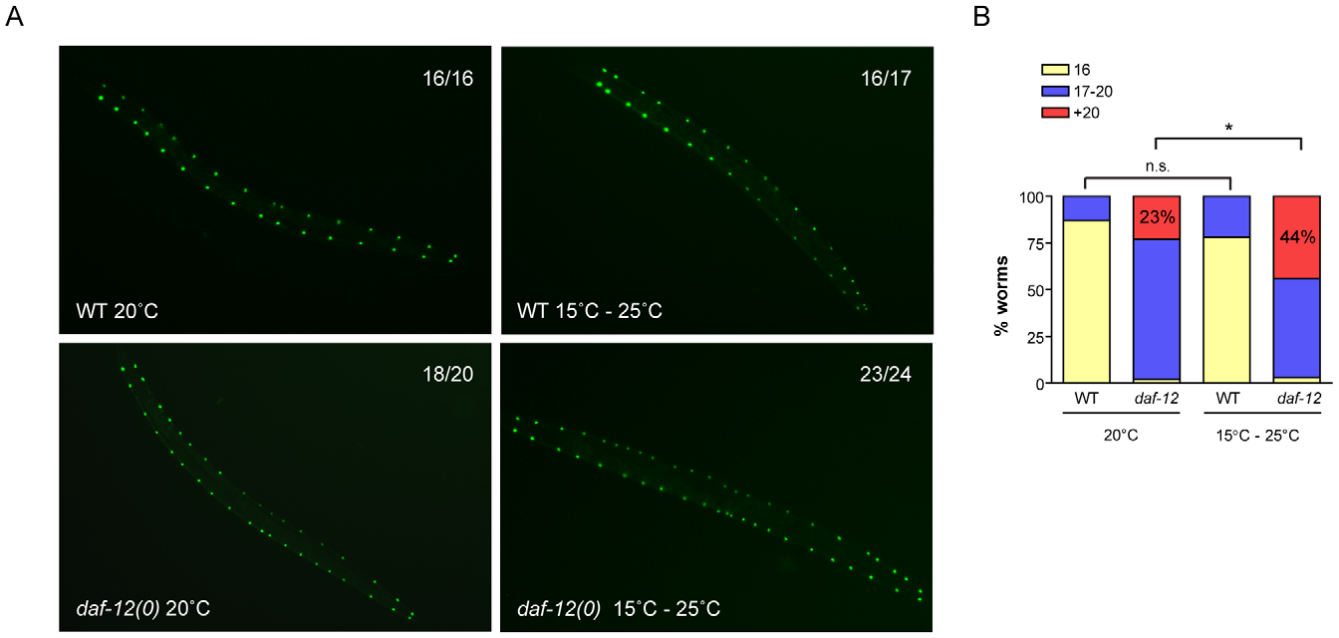
DAF-12 acts as a phenotypic capacitor in worms. (*A*) Images of WT or *daf-12(0)* worms carrying the SCM::GFP transgene that were grown at 20°C or cycles of 15°C–25°C (4 hours each) until adulthood. The number of hypodermal seam cells per side is indicated in each figure. (*B*) Graph showing quantification of no, weak or strong heterochronic phenotypes. Differences were analyzed using Fisher's exact test with SAS 9.2 software. * *p*<0.05.

As *daf-12(0)* worms are not able to buffer genetic mutations among key developmental genes such as miRISC genes, we hypothesized that this nuclear receptor could have a role as a phenotypic capacitor. Phenotypic capacitors act to minimize the effects of either environmental or internal variations of a stereotyped developmental process. To test whether *daf-12* behaves this manner, we exposed WT or *daf-12(0)* worms carrying the SCM::GFP transgene to environmental fluctuations during development. Developing worms were grown for 72 hours at 20°C (control) or in a variable environment consisting of shifts between 15°C and 25°C every 4 hours. As seen in Figure 8A, while WT worms occasionally show an extra hypodermal cell division, the shifts in temperature during development significantly enhance the heterochronic defects exhibited in *daf-12(0)* worms. Interestingly, the number of seam cells in both sides is different, suggesting that environmental fluctuation leads to stochastic errors in seam cell division. When compared *daf-12(0)*, stress conditions almost double the number of worms with strong heterochronic phenotypes (Figure 8A and 8B). Together these results suggest that *daf-12(0)* worms are sensitized to environmental conditions and confirm that DAF-12 has phenotypic capacitor properties.

## Discussion

### Identification of DAF-12 target genes

While DAF-12 is necessary for the decision between reproductive development and dauer formation, target genes necessary for these biological functions are still unidentified. In the present work, we identified putative *in vivo* target genes for DAF-12 in the L2 and adult stages. Several lines of evidence support the specificity and relevance of the binding sites found in our array. First, the highest density of binding sites is found within a 1.5 Kb region upstream of target genes, consistent with DAF-12 often acting by binding to proximal promoter regions. We also found binding sites within genes corresponding to promoter regions of alternative isoforms of the same transcript, such as *din-1S* and *lin-42A*. Second, DAF-12 binding sites overlap with RNA Pol II binding sites annotated in an independent ChIP-seq analysis, suggesting that DAF-12 binding sites correspond to regions of active transcription. Third, two independent strains (ALF4 and ALF9) carrying a TAP-tagged *daf-12*, precipitated either with IgG sepharose or with an anti-DAF-12 antibody, show the same binding regions (Figure 1C and not shown). Fourth, many targets, such as the heterochronic genes *dre-1*, *hbl-1*, *lin-14*, *lin-28* and *lin-66* have been shown to have genetic interactions with *daf-12* [1], [11], [18], [20], [24], [55]. Moreover, known DAF-12 target genes, such *myo-2*, *ceh-22*, *lit-1*, *mir-84* and *mir-241* were identified by our ChIP-chip experiments [18], [33], [37]. Together these results suggest that we have successfully identified *in vivo* targets of DAF-12.

### DAF-12 controls development by a multi-level regulation of gene expression

Our finding that multiple heterochronic genes contain DAF-12 binding sites, suggests that this receptor directly regulates the heterochronic circuit and is consistent with previous observations that the gain of function allele *daf-12(rh61)* shows heterochronic phenotypes [1], [10]. DAF-12 also regulates expression of additional genes with heterochronic phenotypes, such as miRNAs (Figure S7) [18], [19] and components of the miRISC complex [56]. Our results show that among miRISC genes, the nuclear receptor DAF-12 directly regulates at least the *alg-2* and *ain-1* promoters, suggesting that DAF-12 could regulate both miRNA expression and activity.

**Figure 9:**
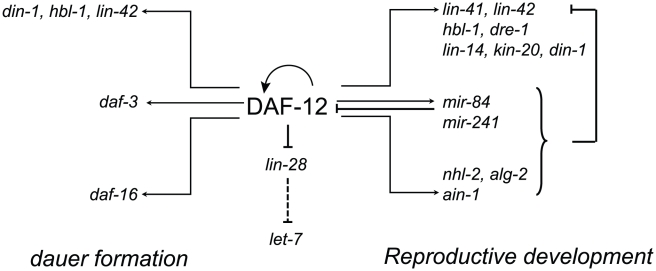
Multi-level control of genes involved in developmental decisions by DAF-12. The nuclear receptor directly regulates the heterochronic circuit, miRNA biogenesis and miRISC expression at the transcriptional level. Other regulators of miRNA activity, such as *lin-28* are repressed by DAF-12. Together the miRNA and miRISC regulate the levels and translation of many heterochronic genes. DAF-12 also promotes dauer entry by regulating dauer loci. Finally, DAF-12 regulates its own expression and is also a target for miRNA.

Beyond effects on miRNA expression and activity, DAF-12 regulates other genes that may control miRNA maturation. Recent evidence in mammals points to a role for LIN-28 in inhibiting *let-7* processing from pri and pre-let-7 [57]. We show here that the RNA-binding gene *lin-28* is repressed by DAF-12 from the L2 to L4 stages. Assuming a conserved function in *C. elegans*, *lin-28* repression by DAF-12 could be required for *let-7* activity to promote execution of L3 programs. We also found the F box gene *dre-1* to be regulated by DAF-12. While no target proteins have been found so far for this ubiquitin ligase, its down-regulation produces heterochronic defects [24], suggesting a key function for DRE-1 in development. Control of *dre-1* by DAF-12 could result in effects on protein stability or degradation. DAF-12 can also regulate its own expression, as well as genes that modulate its activity, such as the co-repressor DIN-1. Our results suggest that DAF-12 regulates gene expression by mechanisms including direct regulation of target promoters, miRNA biogenesis, miRNA activity via miRISC expression and perhaps control of miRNA maturation via *lin-28* (Figure 9).

### DAF-12 acts as a phenotypic capacitor

Fluctuations in environmental conditions and genetic mutations during development can have an impact on the phenotypic outcome of a given organism. How biological systems can reduce the impact of these stochastic perturbations to ensure a uniform phenotype is termed canalization or robustness [58]. Genes who function in part to reduce the effect of environment or genetic variability are called phenotypic capacitors and they contribute to robustness [26]. During reproductive development, our data shows that DAF-12 is required for proper expression of multiple heterochronic genes. While loss of *daf-12* rarely produces penetrant heterochronic phenotypes during development, the phenotypes of many heterochronic genes are augmented by loss of *daf-12* (Figure S4). The lack of a strong null phenotype and enhancement of other genotypes suggests that DAF-12 may act to minimize the developmental consequence of mutations in multiple developmental genes. This action is consistent with a role as a phenotypic capacitor. When conditions become inappropriate for reproductive development, DAF-12 binds to the co-repressor DIN-1 and together this complex represses the heterochronic circuit to enter diapause. Entry into dauer results in many heterochronic phenotypes being suppressed upon dauer recovery (Figure S6). The ability of dauer arrest to suppress the effects of heterochronic mutations is also consistent with a role for DAF-12 as a capacitor.

Besides dauer formation, DAF-12 has been implicated in other phenotypic decisions, such as the development of parasitic larvae [59] or formation of a tooth-like structure in the nematode *P. pacificus* [60]. Phenotypic capacitors such as DAF-12 can play important roles in evolution by buffering new mutations that could otherwise disrupt development and have allow the expression of new phenotypes in adult life [26].

DAF-12 could ensure robustness during reproductive development because it may act as a ‘hub’, interacting with a large number of genes to create a network of mutually regulating genes. Such networks have been shown to be important for robustness [61]. Also, DAF-12 could secure robustness by positive regulation of dauer loci, such as *daf-3*, *daf-16* or *hbl-1* and by repression of genes that inhibit dauer formation, such as *lin-42* [54], [62]. In addition, the system can buffer perturbations because gene products, such as miRNAs or DIN-1, modulated by DAF-12 can also regulate DAF-12 activity (Figure 9) [19].

## Materials and Methods

### Strains

ALF3 strain (*daf-12(rh61rh411), unc-119(ed3)*) was generated by crossing DP38 (*unc-119(ed3)*) and AA86 (*daf-12(rh61rh411)*). The *unc-119* phenotype was verified by behavior and *daf-12* null allele was verified by PCR. AA86, AA82 (*daf-12(rh284)*), AA34 (*daf-12(rh61)*), JR667 (*unc-119(e2498::Tc1)III; wIs51*) and DR1568 (*daf-2(e1371)*) were obtained from the CGC which is supported by NIH funding. MM5438 (*elt-2::GFP, rol-6*) was kindly provided by Dr Morris F. Maduro (UC Riverside).

### Generation of a TAP tagged *daf-12* transgene

The *daf-12:TAP* transgene was generated from the fosmid WRM0611cB06 (Source Bioscience, Nottingham, UK) by homologous recombination in the SW106 *E. coli* strain as previously described [29]. Briefly, in the first step the *galK* gene was inserted into the fosmid between amino acids 26 and 27 in exon 1 using a PCR product generated from pMOD4 *galK*-GT using oligos containing 50 bp regions of fosmid homology (Dataset S1). Then, the *galK* gene was replaced with the TAP tag using a PCR product generated from pBS1761 with the same oligos [63]. The *unc-119* marker was then added to this fosmid via *cre-loxP* recombination with pLoxP *unc-119* [29]. The EPI300 strain (Epicentre Biotechnologies) was then used to amplify the fosmid for purification with the FosmidMAX DNA purification kit (Epicentre). The modified fosmid was then sequenced to verify the tag sequence and location.

### Transgenic worms

ALF4 and ALF9 strains were obtained by biolistic bombardment of ALF3 with the TAP-tagged *daf-12* fosmid as previously described [64]. ALF62 and ALF63 strains were generated by biolistic bombardment of the ALF3 strain. ALF62 (*unc-119 (ed3), bafIs62 (lin-42p::GFP)*) was generated by integration of an extra chromosomal array by TMP/UV irradiation [65]. ALF63 (*unc-119 (ed3), bafIs63 (lin-42pmut::GFP)*) was directly obtained from bombardment. ALF62 and ALF63 were then crossed with HT1593 (*unc-119(ed3)*) to generate *daf-12*(+) strains. ALF70 (*unc-119 (ed3), bafIs70 (alg-2p::GFP)*) and ALF72 (*unc-119 (ed3), bafIs70 (ain-1p::GFP)*) were generated by biolistic bombardment of the DP38 strain (*unc-119(ed3)*). These were crossed with ALF3 to place the reporters in a *daf-12(0)* background (ALF73 and ALF74 respectively).

### Sample preparation and ChIP-chip

Synchronized worms at mid-L2 stage and young adults were collected by floatation, rinsed with milliQ water (DEPC treated) and frozen at −80°C. Whole *C. elegans* were simultaneously broken up and fixed by brief sonication in PBS containing 1% formaldehyde. Total fixation time was 30 minutes at room temperature, after which the reaction was quenched by the addition of glycine to a final concentration of 0.125 M. Preparation of chromatin and ChIP was carried out essentially as described [66]. In brief, lysates were sonicated and the DNA sheared to an average length of 300–500 bp. Genomic DNA (Input) was prepared by treating aliquots of chromatin with RNase, proteinase K and heat for de-crosslinking, followed by phenol/chloroform extraction and ethanol precipitation. Pellets were resuspended and the resulting DNA was quantified on a Nanodrop spectrophotometer. Extrapolation to the original chromatin volume allowed quantification of the total chromatin yield.

Equal amounts of L2 and adult chromatin (20 µg each) were mixed together and samples were pre-cleared with protein A agarose beads (Invitrogen). Genomic DNA regions of interest were isolated using an antibody against DAF-12 (Santa Cruz, ce-267). After incubation at 4°C overnight, protein A agarose beads were used to isolate the immune complexes. Complexes were washed, eluted from the beads with SDS buffer, and subjected to RNase and proteinase K treatment. Crosslinks were reversed by incubation overnight at 65°C, and ChIP DNA was purified by phenol-chloroform extraction and ethanol precipitation.

Quantitative PCR (QPCR) reactions were carried out in triplicate on specific genomic regions using SYBR Green Supermix (Bio-Rad), site-specific primer pairs, and an aliquot of ChIP DNA. The resulting signals were normalized for primer efficiency by carrying out QPCR for each primer pair using Input DNA.

ChIP and Input DNAs were amplified by whole-genome amplification (WGA) using the GenomePlex WGA Kit (Sigma). The resulting amplified DNAs were purified, quantified, and tested by QPCR at the same specific genomic regions as the original ChIP DNA to assess quality of the amplification reactions. Amplified DNAs were fragmented and labeled using the DNA Terminal Labeling Kit from Affymetrix, and then hybridized to Affymetrix GeneChip *C. elegans* Tiling 1.0R arrays at 45°C overnight. Arrays were washed and scanned, and the resulting CEL files were analyzed using Affymetrix TAS software that computed for each probe estimates of fold enrichment (in linear scale) over hybridization with input DNA. At the same time, TAS calculated for each probe a p-value by applying a Wilcoxon signed rank test. A threshold of 2.5 was selected, which corresponds to probe intensities approximately 2.5× stronger on the ChIP array than on the Input array. Additional TAS threshold parameters were MinRun = 180 bp, MaxGap = 300 bp. TAS analysis showed that the selected threshold of 2.5 corresponds approximately to a p-value of 0.01. In addition, the false discovery rate (FDR) was estimated as previously described [67]. At a threshold of 2.5, the FDRs for the 4 samples were estimated to be: AA86 = 3.17%, ALF4(1) = 0.25%, ALF4(2) = 0.34% and ALF4(3) = 2.33%. The TAS-generated BED files (containing the peak intervals) were analyzed using Genpathway (now Active Motif) proprietary software that provides comprehensive information on genomic annotation, peak metrics and sample comparisons for all peaks. Graphs of genomic regions with peak enrichment values were generated using the Affymetrix Integrated Genome Browser. The data discussed in this publication have been deposited in NCBI's Gene Expression Omnibus [31] and are accessible through GEO Series accession number GSE28350 (http://www.ncbi.nlm.nih.gov/geo/query/acc.cgi?acc=GSE28350).

### Western blotting

Protein was extracted from N2, ALF3, AA86 or transgenic worms by boiling in LDS loading buffer (Invitrogen). After sonication, samples were run on a 10% polyacrylamide gel and transferred to PVDF. Membranes were probed with 1∶750 α-DAF-12 (ce-267, Santa Cruz), and 1∶3000 actin (sc-1616R, Santa Cruz) followed by IRDye 680 goat anti-rabbit (1∶10000) (Li-Cor Biosciences). For detection of DAF-12 expressed in HEK cells, α-myc (9E10, Covance) was used at a dilution of 1∶1000.

### Computational analysis

Using the Affymetrix TAS software (Affymetrix), we identified all genes associated with probes that were positive in transgenic Chromatin IPs, but negative in the control and plotted the distance between the bound probe and the ATG proximal to each gene. We next downloaded available GO annotations for all genes associated with bound probes (WormMart, 04/09/2010) and classified them using the GO.db library (Version 2.2.0) in R (http://www.r-project.org/). To find de novo DAF-12 binding sites, we identified all sequence intervals associated with genes that are active in experimental samples, but not in the control. We submitted 600 unique sequences of 100 nt each, centered on the strongest DAF-12 binding signals to MEME (http://meme.mbcr.net) after masking repeat sequences using RepeatMasker (http://www.repeatmasker.org). As a negative control, we also likewise analyzed 600 unique sequences of 100 nt each from intervals with the weakest DAF-12 binding signals associated with genes that did not score positive in the control or experimental samples 1 and 2.

### RNA preparation, RT-qPCR, and NanoString analysis

Eggs from N2 and mutant strains were obtained by hypochlorite treatment and hatched overnight in S-Basal with shaking. The arrested L1 larvae were put on NGA plates to resume development. Samples were collected by floatation at the indicated developmental stages, rinsed in milliQ water and snap-frozen. Dauer larvae were isolated by a 15% Ficoll 400, 0.1 M NaCl gradient. RNA extraction and qPCR was performed as previously described [68]. For Nanostring analysis, RNA samples from 6 worm collections were analyzed by Expression Analysis (Durham, NC).

### Plasmid DNAs

The DAF-12 A1 cDNA was PCR amplified from *C. elegans* cDNA and subcloned as a SfiI-BglI fragment into pCMV myc (Clonetech). The DAF-12 *rh284* mutant was made with the Quick-Change mutagenesis kit (Stratagene). For luciferase reporter genes, fragments between 2 Kb and 3.5 Kb upstream the ATG were PCR amplified and subcloned into pGL3 Luc Promoter (Promega). For GFP reporter strains, promoter regions were subcloned in pPD95.75 vector (A gift of Dr. Andy Fire). Oligos sequences are available in Figure S8. The *unc-119* rescue fragment was introduced by recombination as previously described [69]. The *lin-42::GFP* promoter mutant was synthesized and subcloned into pPD95.75 vector (Celtek Bioscience).

### Cell culture and reporter assay

HEK 293T cells were cultured and transfected (Fugene, Roche) in 24well plates, with DMEM and 10% charcoal FBS treated (Hyclone). Briefly, 225 ng of either pCMV myc DAF-12 A1 or pCMV myc vector were co-transfected with 12.5 ng of pRL-TK (Renilla luciferase) and 25 ng pGL3 Luc promoter with the promoter of interest (Promega). The following day, cells were stimulated with 0.5 µM (25R)-Δ7-DA [70] or DMSO for 16 hr. At this concentration, no difference in activity was observed between the (25R)- Δ7-DA and (25S)- Δ7-DA [71] (not shown). Luciferase was measured using Dual-Luciferase Reporter Assay System (Promega). Each experiment was repeated three times and fold activation is relative to the non stimulated control transfected with pCMV vector, unless indicated otherwise.

### Digital imaging

Transgenic worms were mounted for digital photography using an Olympus BX51 upright microscope. All photos within a panel were taken on the same day with identical camera settings to allow direct comparison. Quantification of the GFP reporters was performed with image J software and analyzed with an unpaired t-test.

### RNAi experiments

L3 worms were transferred to RNAi plates at 16°C for approximately 72 hr. 10 adults were transferred to new RNAi plates and left for 3 hr to lay eggs before being removed. Analysis was performed with the adult F1 progeny.

### Environmental stress

Eggs from WT or *daf-12(0)* worms carrying the SCM::GFP transgene were obtained by hypochlorite treatment and incubated for 72 hours at 20°C or 15°C–25°C cycles of 4 hours each with a programmable incubator (Innova 4230, New Brunswick Scientific, New Brunswick, NJ).

## Supporting Information

Dataset S1 (PDF)

Figure S1 Enriched DAF-12 binding sites were analyzed by (A) DREME or (B) MEME.
(PDF)

Figure S2 Comparison between PHA-4 and DAF-12 target genes.
(EPS)

Figure S3 DAF-12 binding sites at the *lin-42* locus.
(EPS)

Figure S4 Heterochronic phenotypes enhanced by *daf-12(0)*.
(PDF)

Figure S5 Mutation of M-2 and DR domains (highlighted) within *lin-42A* promoter. Mutations are shown above the WT sequence.
(PDF)

Figure S6 Heterochronic phenotypes suppressed by dauer formation.
(PDF)

Figure S7 Table of DAF-12 target miRNAs.
(PDF)

Figure S8 Oligos used to subclone promoter regions for pGL3 Luc and pPD95.75 vectors. Restriction sites added for subcloning are indicated in red.
(PDF)

Table S1 Genes within 1 Kb, 2 Kb and 5 Kb of identified DAF-12 binding sites.
(XLS)

Table S2 Genes identified as DAF-12 binding sites when control (*daf-12(0)*) was positive.
(XLS)

Table S3 Gene ontology analysis of DAF-12 targets with DAVID program.
(XLS)

## References

[1] Antebi A, Culotti JG, Hedgecock EM (1998). daf-12 regulates developmental age and the dauer alternative in Caenorhabditis elegans.. Development.

[2] Cassada RC, Russell RL (1975). The dauerlarva, a post-embryonic developmental variant of the nematode Caenorhabditis elegans.. Dev Biol.

[3] Riddle DL, Swanson MM, Albert PS (1981). Interacting genes in nematode dauer larva formation.. Nature.

[4] Gill MS, Held JM, Fisher AL, Gibson BW, Lithgow GJ (2004). Lipophilic regulator of a developmental switch in Caenorhabditis elegans.. Aging Cell.

[5] Held JM, White MP, Fisher AL, Gibson BW, Lithgow GJ (2006). DAF-12-dependent rescue of dauer formation in Caenorhabditis elegans by (25S)-cholestenoic acid.. Aging Cell.

[6] Motola DL, Cummins CL, Rottiers V, Sharma KK, Li T (2006). Identification of ligands for DAF-12 that govern dauer formation and reproduction in C. elegans.. Cell.

[7] Gerisch B, Weitzel C, Kober-Eisermann C, Rottiers V, Antebi A (2001). A hormonal signaling pathway influencing C. elegans metabolism, reproductive development, and life span.. Dev Cell.

[8] Jia K, Albert PS, Riddle DL (2002). DAF-9, a cytochrome P450 regulating C. elegans larval development and adult longevity.. Development.

[9] Rottiers V, Motola DL, Gerisch B, Cummins CL, Nishiwaki K (2006). Hormonal control of C. elegans dauer formation and life span by a Rieske-like oxygenase.. Dev Cell.

[10] Antebi A, Yeh WH, Tait D, Hedgecock EM, Riddle DL (2000). daf-12 encodes a nuclear receptor that regulates the dauer diapause and developmental age in C. elegans.. Genes Dev.

[11] Ludewig AH, Kober-Eisermann C, Weitzel C, Bethke A, Neubert K (2004). A novel nuclear receptor/coregulator complex controls C. elegans lipid metabolism, larval development, and aging.. Genes Dev.

[12] Fisher AL, Lithgow GJ (2006). The nuclear hormone receptor DAF-12 has opposing effects on Caenorhabditis elegans lifespan and regulates genes repressed in multiple long-lived worms.. Aging Cell.

[13] Gems D, Sutton AJ, Sundermeyer ML, Albert PS, King KV (1998). Two pleiotropic classes of daf-2 mutation affect larval arrest, adult behavior, reproduction and longevity in Caenorhabditis elegans.. Genetics.

[14] Larsen PL, Albert PS, Riddle DL (1995). Genes that regulate both development and longevity in Caenorhabditis elegans.. Genetics.

[15] Gerisch B, Rottiers V, Li D, Motola DL, Cummins CL (2007). A bile acid-like steroid modulates Caenorhabditis elegans lifespan through nuclear receptor signaling.. Proc Natl Acad Sci U S A.

[16] Hsin H, Kenyon C (1999). Signals from the reproductive system regulate the lifespan of C. elegans.. Nature.

[17] Ambros V (1989). A hierarchy of regulatory genes controls a larva-to-adult developmental switch in C. elegans.. Cell.

[18] Bethke A, Fielenbach N, Wang Z, Mangelsdorf DJ, Antebi A (2009). Nuclear hormone receptor regulation of microRNAs controls developmental progression.. Science.

[19] Hammell CM, Karp X, Ambros V (2009). A feedback circuit involving let-7-family miRNAs and DAF-12 integrates environmental signals and developmental timing in Caenorhabditis elegans.. Proc Natl Acad Sci U S A.

[20] Abbott AL, Alvarez-Saavedra E, Miska EA, Lau NC, Bartel DP (2005). The let-7 MicroRNA family members mir-48, mir-84, and mir-241 function together to regulate developmental timing in Caenorhabditis elegans.. Dev Cell.

[21] Reinhart BJ, Slack FJ, Basson M, Pasquinelli AE, Bettinger JC (2000). The 21-nucleotide let-7 RNA regulates developmental timing in Caenorhabditis elegans.. Nature.

[22] Abrahante JE, Daul AL, Li M, Volk ML, Tennessen JM (2003). The Caenorhabditis elegans hunchback-like gene lin-57/hbl-1 controls developmental time and is regulated by microRNAs.. Dev Cell.

[23] Abrahante JE, Miller EA, Rougvie AE (1998). Identification of heterochronic mutants in Caenorhabditis elegans. Temporal misexpression of a collagen::green fluorescent protein fusion gene.. Genetics.

[24] Fielenbach N, Guardavaccaro D, Neubert K, Chan T, Li D (2007). DRE-1: an evolutionarily conserved F box protein that regulates C. elegans developmental age.. Dev Cell.

[25] Liu Z, Ambros V (1991). Alternative temporal control systems for hypodermal cell differentiation in Caenorhabditis elegans.. Nature.

[26] Masel J, Siegal ML (2009). Robustness: mechanisms and consequences.. Trends Genet.

[27] Rigaut G, Shevchenko A, Rutz B, Wilm M, Mann M (1999). A generic protein purification method for protein complex characterization and proteome exploration.. Nat Biotechnol.

[28] Gottschalk A, Almedom RB, Schedletzky T, Anderson SD, Yates JR (2005). Identification and characterization of novel nicotinic receptor-associated proteins in Caenorhabditis elegans.. EMBO J.

[29] Zhang Y, Nash L, Fisher AL (2008). A simplified, robust, and streamlined procedure for the production of C. elegans transgenes via recombineering.. BMC Dev Biol.

[30] Rottiers V, Antebi A (2006). Control of Caenorhabditis elegans life history by nuclear receptor signal transduction.. Exp Gerontol.

[31] Edgar R, Domrachev M, Lash AE (2002). Gene Expression Omnibus: NCBI gene expression and hybridization array data repository.. Nucleic Acids Res.

[32] Zhong M, Niu W, Lu ZJ, Sarov M, Murray JI (2010). Genome-wide identification of binding sites defines distinct functions for Caenorhabditis elegans PHA-4/FOXA in development and environmental response.. PLoS Genet.

[33] Shostak Y, Van Gilst MR, Antebi A, Yamamoto KR (2004). Identification of C. elegans DAF-12-binding sites, response elements, and target genes.. Genes Dev.

[34] Chatterjee S, Grosshans H (2009). Active turnover modulates mature microRNA activity in Caenorhabditis elegans.. Nature.

[35] Hammell CM, Lubin I, Boag PR, Blackwell TK, Ambros V (2009). nhl-2 Modulates microRNA activity in Caenorhabditis elegans.. Cell.

[36] Huang da W, Sherman BT, Lempicki RA (2009). Systematic and integrative analysis of large gene lists using DAVID bioinformatics resources.. Nat Protoc.

[37] Ao W, Gaudet J, Kent WJ, Muttumu S, Mango SE (2004). Environmentally induced foregut remodeling by PHA-4/FoxA and DAF-12/NHR.. Science.

[38] Bailey TL, Williams N, Misleh C, Li WW (2006). MEME: discovering and analyzing DNA and protein sequence motifs.. Nucleic Acids Res.

[39] Musgrove EA, Sutherland RL (2009). Biological determinants of endocrine resistance in breast cancer.. Nat Rev Cancer.

[40] Masuyama H, Brownfield CM, St-Arnaud R, MacDonald PN (1997). Evidence for ligand-dependent intramolecular folding of the AF-2 domain in vitamin D receptor-activated transcription and coactivator interaction.. Mol Endocrinol.

[41] Nasrin N, Ogg S, Cahill CM, Biggs W, Nui S (2000). DAF-16 recruits the CREB-binding protein coactivator complex to the insulin-like growth factor binding protein 1 promoter in HepG2 cells.. Proc Natl Acad Sci U S A.

[42] Paradis S, Ailion M, Toker A, Thomas JH, Ruvkun G (1999). A PDK1 homolog is necessary and sufficient to transduce AGE-1 PI3 kinase signals that regulate diapause in Caenorhabditis elegans.. Genes Dev.

[43] Moss EG (2007). Heterochronic genes and the nature of developmental time.. Curr Biol.

[44] Slack FJ, Basson M, Liu Z, Ambros V, Horvitz HR (2000). The lin-41 RBCC gene acts in the C. elegans heterochronic pathway between the let-7 regulatory RNA and the LIN-29 transcription factor.. Mol Cell.

[45] Moss EG, Lee RC, Ambros V (1997). The cold shock domain protein LIN-28 controls developmental timing in C. elegans and is regulated by the lin-4 RNA.. Cell.

[46] Banerjee D, Kwok A, Lin SY, Slack FJ (2005). Developmental timing in C. elegans is regulated by kin-20 and tim-1, homologs of core circadian clock genes.. Dev Cell.

[47] Jeon M, Gardner HF, Miller EA, Deshler J, Rougvie AE (1999). Similarity of the C. elegans developmental timing protein LIN-42 to circadian rhythm proteins.. Science.

[48] Coller J, Parker R (2005). General translational repression by activators of mRNA decapping.. Cell.

[49] Geiss GK, Bumgarner RE, Birditt B, Dahl T, Dowidar N (2008). Direct multiplexed measurement of gene expression with color-coded probe pairs.. Nat Biotechnol.

[50] Rutherford SL, Lindquist S (1998). Hsp90 as a capacitor for morphological evolution.. Nature.

[51] Simmer F, Tijsterman M, Parrish S, Koushika SP, Nonet ML (2002). Loss of the putative RNA-directed RNA polymerase RRF-3 makes C. elegans hypersensitive to RNAi.. Curr Biol.

[52] Rougvie AE (2001). Control of developmental timing in animals.. Nat Rev Genet.

[53] Lambie EJ (2002). Cell proliferation and growth in C. elegans.. Bioessays.

[54] Tennessen JM, Opperman KJ, Rougvie AE (2010). The C. elegans developmental timing protein LIN-42 regulates diapause in response to environmental cues.. Development.

[55] Morita K, Han M (2006). Multiple mechanisms are involved in regulating the expression of the developmental timing regulator lin-28 in Caenorhabditis elegans.. EMBO J.

[56] Grishok A, Pasquinelli AE, Conte D, Li N, Parrish S (2001). Genes and mechanisms related to RNA interference regulate expression of the small temporal RNAs that control C. elegans developmental timing.. Cell.

[57] Viswanathan SR, Daley GQ, Gregory RI (2008). Selective blockade of microRNA processing by Lin28.. Science.

[58] Waddington CH (1959). Canalization of development and genetic assimilation of acquired characters.. Nature.

[59] Wang Z, Zhou XE, Motola DL, Gao X, Suino-Powell K (2009). Identification of the nuclear receptor DAF-12 as a therapeutic target in parasitic nematodes.. Proc Natl Acad Sci U S A.

[60] Bento G, Ogawa A, Sommer RJ (2010). Co-option of the hormone-signalling module dafachronic acid-DAF-12 in nematode evolution.. Nature.

[61] Lehner B, Crombie C, Tischler J, Fortunato A, Fraser AG (2006). Systematic mapping of genetic interactions in Caenorhabditis elegans identifies common modifiers of diverse signaling pathways.. Nat Genet.

[62] Karp X, Ambros V (2011). The Developmental Timing Regulator hbl-1 Modulates the Dauer Formation Decision in Caenorhabditis elegans.. Genetics.

[63] Puig O, Caspary F, Rigaut G, Rutz B, Bouveret E (2001). The tandem affinity purification (TAP) method: a general procedure of protein complex purification.. Methods.

[64] Hochbaum D, Ferguson AA, Fisher AL (2010). Generation of transgenic C. elegans by biolistic transformation.. J Vis Exp.

[65] Gengyo-Ando K, Mitani S (2000). Characterization of mutations induced by ethyl methanesulfonate, UV, and trimethylpsoralen in the nematode Caenorhabditis elegans.. Biochem Biophys Res Commun.

[66] Soutoglou E, Talianidis I (2002). Coordination of PIC assembly and chromatin remodeling during differentiation-induced gene activation.. Science.

[67] Johnson WE, Li W, Meyer CA, Gottardo R, Carroll JS (2006). Model-based analysis of tiling-arrays for ChIP-chip.. Proc Natl Acad Sci U S A.

[68] Ferguson AA, Springer MG, Fisher AL (2010). skn-1-Dependent and -independent regulation of aip-1 expression following metabolic stress in Caenorhabditis elegans.. Mol Cell Biol.

[69] Ferguson AA, Fisher AL (2009). Retrofitting ampicillin resistant vectors by recombination for use in generating C. elegans transgenic animals by bombardment.. Plasmid.

[70] Martin R, Däbritz F, Entchev EV, Kurzchalia TV, Knölker H-J (2008). Stereoselective synthesis of the hormonally active (25S)-delta7-dafachronic acid, (25S)-delta4-dafachronic acid, (25S)-dafachronic acid, and (25S)-cholestenoic acid.. Org Biomol Chem.

[71] Martin R, Schmidt AW, Theumer G, Krause T, Entchev EV (2009). Synthesis and biological activity of the (25R)-cholesten-26-oic acids–ligands for the hormonal receptor DAF-12 in Caenorhabditis elegans.. Org Biomol Chem.

