# Supplementary material for: DAF-12 Regulates a Connected Network of Genes to Ensure Robust Developmental Decisions
Source: PLoS Genet. 2011 Jul 21;7(7):e1002179. doi: 10.1371/journal.pgen.1002179 (PMC3140985; doi:10.1371/journal.pgen.1002179)
Supplement: Dataset S1 — (PDF) [file pgen.1002179.s001.pdf]

DAF-12 TAP F: GGAAATCGAAACAAATGAAAACCCAGATAAGGTAGAGGAGATGGCAGGCCTTGCGC

DAF-12 TAP R: CACTTACCCTGCGGGTTACTCGTTTCCTTCTCACGACGGGAAGCTTATCGTCATCATCAAGTGCC
