# Supplementary figures and images for: DAF-12 Regulates a Connected Network of Genes to Ensure Robust Developmental Decisions
Source: PLoS Genet. 2011 Jul 21;7(7):e1002179. doi: 10.1371/journal.pgen.1002179 (PMC3140985; doi:10.1371/journal.pgen.1002179)

A

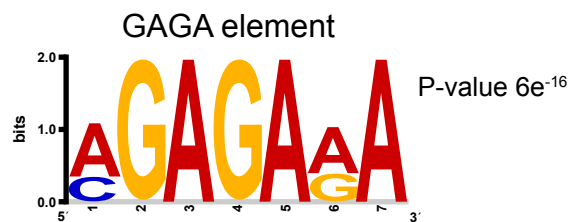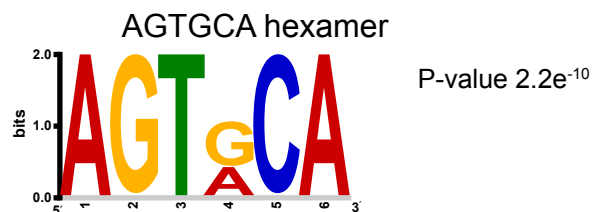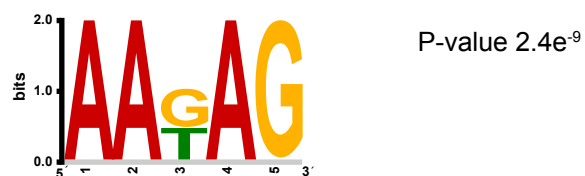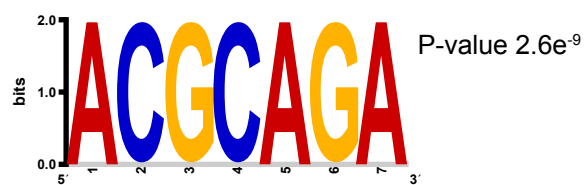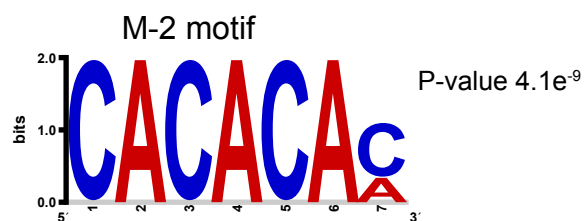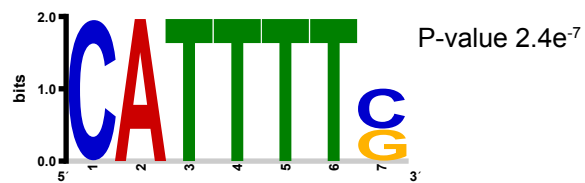

B

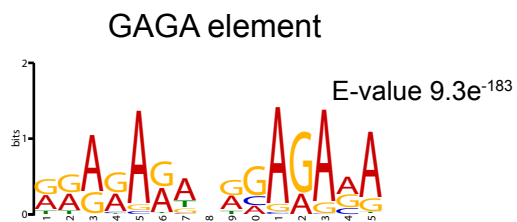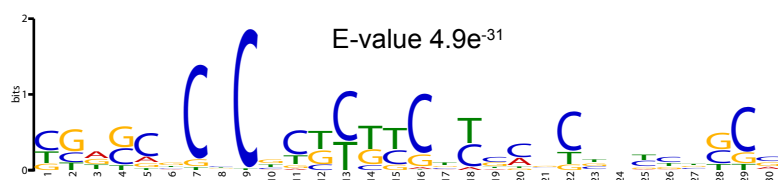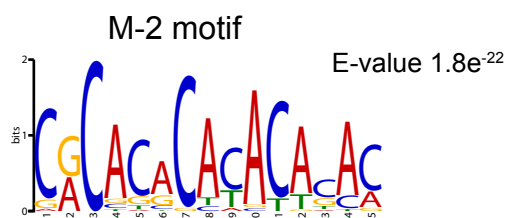

Supplement: Figure S1 — Enriched DAF-12 binding sites were analyzed by (A) DREME or (B) MEME. (PDF) [file pgen.1002179.s002.pdf]
