# Supplementary material for: DAF-12 Regulates a Connected Network of Genes to Ensure Robust Developmental Decisions
Source: PLoS Genet. 2011 Jul 21;7(7):e1002179. doi: 10.1371/journal.pgen.1002179 (PMC3140985; doi:10.1371/journal.pgen.1002179)
Supplement: Figure S4 — Heterochronic phenotypes enhanced by daf-12(0). (PDF) [file pgen.1002179.s005.pdf]

| Gene | Phenotype | Reference |
|---|---|---|
| *dre-1* | Gonad migration | (Fielenbach et al., 2007) |
| *lin-46* | Absence of alae at L4 | (Hammell et al., 2009) |
| *lin-66* | Extra seam cell divisions | (Morita and Han, 2006) |
| *ain-1* | Extra seam cell divisions, Protruding and Burst Vulva, extra intestinal cell divisions | This manuscript |
| *ain-2* | Extra seam cell divisions, synergistic effect on body size | This manuscript |
| *alg-2* | Extra seam cell divisions, extra intestinal cell divisions | This manuscript |
| *cgh-1* | Gaps in alae at L4 | (Hammell et al., 2009) |
| *nhl-2* | Gaps in alae at L4 | (Hammell et al., 2009) |
| *nhl-2* | Extra seam cell divisions, extra intestinal cell divisions, synergistic effect on body size | This manuscript |
