# Supplementary material for: DAF-12 Regulates a Connected Network of Genes to Ensure Robust Developmental Decisions
Source: PLoS Genet. 2011 Jul 21;7(7):e1002179. doi: 10.1371/journal.pgen.1002179 (PMC3140985; doi:10.1371/journal.pgen.1002179)
Supplement: Figure S5 — Mutation of M-2 and DR domains (highlighted) within lin-42A promoter. Mutations are shown above the WT sequence. (PDF) [file pgen.1002179.s006.pdf]

```
   1 TTGGTCGACA TTCCCCCTGA AAAGCATCTC TGAAGTGCCT GAAACTTGAG
  51 AATTTTCCTA CAATTTAAGG TTTTGGACGT TTTTCCAGGG TCCCAAAACA
 101 CGGTGGAATG GTCTGAAACA TGGTGCATCT GAGTATAAAA CGTGGAAATT
 151 CACTCGAAAT TCTAGAGTTT TTTCCGCATT AAATTAAGGG TAGGAGCGGT
 201 CTAGATCATG GTGCATCGAA AACTCCTTGC TAGCCTATCT GTCACCAATT
 251 TTAGGCCAAT ATAGTCCTAT TCAGGCCCCA AGCCCTTCAA ACCACCACCG
 301 CTAAACCTTT TGCAACATCT CCCCCTACCA CCCTCCAACG TCATCCCGCC
                                                  T  T
 351 GGCCATCACG ACGGCCGGCA CGGCGCCAAG AGTACATCTG CATCTCGGAA
       T  T
 401 TAGTGCATGA AAGGGCATCA TCGCTGACTC TCGCCCCCCT CTCTCTCTCC
 451 CGTCCCAAGT GCCAGCCGCG GCAGCGACAG CGGGAGAAAG GGCACCGGCA
 501 GTGGGTAGCG CCGTCGCCGC AGCCGCGGGC TGTCCGCGGC TGGTCGCGCC
                                                     A  A
 551 CGTCCACACT GCCCTGCAGC AGTCTGCGTC TCCTCTCTGG AAGTGTCCTG
                 GAATCC
 601 GCGGCTGAGA GTGTGTGTAT GTGATAGTGA CCATAAGGTG CACCAGGCGA
 651 CCCTAGGCCT TCGGGCTTAG GCCCTGAAAG CCTTCTTATG GCCCTATACT
 701 CCCTATATTC CCTAATCCCG AGCAACTCCA AATGTATGTG TATACCGTAT
 751 CTCAATTATT GAACCAAATT CGGCACCAAC TAATACTCTA CTACTACCTC
                     GGATT C
 801 CACATACTAA CCAAACACAC ATAAAGTCAA ATTTCAAGAG GGTGGATGAT
           A  A
 851 GCTTCTGCAC TTCTCAAGCC CATTAAAGGA TAAGTTTTTA AGCTTAACTT
 901 AATGTTAGTA GGGCTTTTAC TTCCTCATTT GATGTTTAGT TTTGCCATAG
                                                      T  T
 951 GGAGTAGTGT TCGTTGCGCG AAATTGCGTC ACAATAGTCA ATAAAGTACA
1001 AAAACTTCCG TGTTTGCTTA GATCCTTCCC CATGTGAGAA TGCTCATCCC
1051 TCACGGGCTT TGGCTTTTTT GAGATAGTGA AGCTAGACTA GGCCTATCCT
1101 GAGCCCAAAA ACCTGGGCTC TTATGGCAGT AGACCCTCCC TAAGGGCACT
                            T  T
1151 GGCTAGACCT AAACATACCT AGGACATGCG CCTTAAAAGC CTACTCCTAG
1201 GAGAGAAGGT CTGGTCCTAA GGGGCCTAAT CGTTAAGGGC CTGGCCCCAA
1251 GTGGATTGGC CCTGGTCCTT ATGGGTTACA ACTTTATAGG CCTAGTCCTT
1301 GAAGACCTGA TTATTAGGGC CCTGGTCCCT AGAGCCCCGG ACCTTTCAGC
1351 CTGCATCTAA AAGACCCGGT CCTTGCGTCA GGGGCCTGGT CCAAAGGGGC
1401 TGGTTTGAGG GGGCCTGGGT CTTAGGGGTC CGGTCCAAAA GGACCCGTTC
```

1451 TCAGGGCCCG GTTCTAGAGG ACCTAGTTCT AGAAGACTCG GTCTTTATGG

1501 GTCTGGGCCT AACCCCCTGA CCCCCCAGCT TTAGCTCCTA GGTAACCGGT

1551 CATAAGGGGC TTGGTGGTTA CTCCCTAAGG GTTAAAACTT TATAGGCCTG

1601 TTCCTTAAAA ACCTGATCAT TAGGCCCCTG GTTCTAGAGC CCCAGTCCCT

1651 TTTGCCTGGA TCTGGAGGGC CCGGTGCTTA GGGGCCTGAT CCTAAGAAAT

1701 CTGGCCCCAG CTTCAGCTCC TAGGTAACCC TGCAAAAGTG CTCCTCTTTA

T   T

1751 TTTTTCTTCT TCTTCTTCCT ACTGCGACCT TTTGACAATA GTACACACAG

1801 ACAGTCACAA CAAACACCCC TCCCTTCCCA CCCAGCCGGG TAGCCCCCCG

1851 TGCCAGCCAT CCTTACACCG GCTGCGTCTC CCGTAGCCCG ACGCGCTCTC

1901 TCGTTTCTCC CGCACGCTGC GCGTCCAACA GGGTTCCCGT ACCTGTCTCC

1951 TCGACCCCAA CCAAGACAGG CCGCCCCTAT TATTCTCACA TCTTGCCATC

2001 ATCACCACCA CTACCAGTCT GTCCAACTGA CCAGTACCCC CTTACCTAGA

2051 AACTCCTAAG CCTACCACCC TAAAGGATCC TTT
