# Supplementary material for: DAF-12 Regulates a Connected Network of Genes to Ensure Robust Developmental Decisions
Source: PLoS Genet. 2011 Jul 21;7(7):e1002179. doi: 10.1371/journal.pgen.1002179 (PMC3140985; doi:10.1371/journal.pgen.1002179)
Supplement: Figure S6 — Heterochronic phenotypes suppressed by dauer formation. (PDF) [file pgen.1002179.s007.pdf]

Gene	Phenotype suppressed by dauer formation	Reference
daf-12	Gonad migration and late stage phenotypes	(Antebi et al. 1998)
dre-1	Precocious seam cells fusion and alae formation, gaps in adult alae	(Fielenbach et al. 2007)
hbl-1	Seam cell division and precocious fusion<br>Vulval phenotypes	(Abrahante et al. 2003)
lin-4	Retarded alae formation	(Liu and Ambros 1991)
lin-14	Precocious or retarded alae formation	(Liu and Ambros 1991)
lin-28	Precociously alae formation	(Liu and Ambros 1991)
lin-42	Precociously alae formation	(Abrahante et al. 1998)
lin-58	Precociously alae formation	(Abrahante et al. 1998)
