# Supplementary material for: DAF-12 Regulates a Connected Network of Genes to Ensure Robust Developmental Decisions
Source: PLoS Genet. 2011 Jul 21;7(7):e1002179. doi: 10.1371/journal.pgen.1002179 (PMC3140985; doi:10.1371/journal.pgen.1002179)
Supplement: Figure S7 — Table of DAF-12 target miRNAs. (PDF) [file pgen.1002179.s008.pdf]

Transcript	Name
M04C9.7	mir-2
T12E12.7	mir-228
F46G11.5	mir-230
F22F1.4	mir-237
C34E11.5	mir-239.1
C34E11.6	mir-239.2
F56A12.4	mir-241
F36A4.16	mir-242
R08C7.14	mir-243
ZK593.10	mir-246
F44E7.11	mir-253
F25D1.6	mir-259
C06H2.8	mir-268
Y41G9A.7	mir-34
Y62F5A.2	mir-35
D1007.17	mir-353
Y105E8A.31	mir-354
ZK652.12	mir-356
Y62F5A.3	mir-36
Y62F5A.4	mir-37
Y62F5A.5	mir-38
Y62F5A.6	mir-39
F54B11.12	mir-392
Y62F5A.7	mir-40
Y62F5A.8	mir-41
F36H1.7	mir-51
Y37A1B.16	mir-52
F36H1.8	mir-53
F09A5.5	mir-54
F09A5.6	mir-55
Y67D8A.4	mir-58
C32D5.13	mir-60
F16A11.4	mir-71
F53G2.9	mir-72
T24D8.9	mir-73
T24D8.7	mir-74
T24D8.8	mir-75
C06A6.6	mir-83
B0395.4	mir-84
K01F9.1	mir-90
