# Supplementary material for: DAF-12 Regulates a Connected Network of Genes to Ensure Robust Developmental Decisions
Source: PLoS Genet. 2011 Jul 21;7(7):e1002179. doi: 10.1371/journal.pgen.1002179 (PMC3140985; doi:10.1371/journal.pgen.1002179)
Supplement: Figure S8 — Oligos used to subclone promoter regions for pGL3 Luc and pPD95.75 vectors. Restriction sites added for subcloning are indicated in red. (PDF) [file pgen.1002179.s009.pdf]

Gene	Forward primer	Reverse primer
ain-1	ATAGCTAGCGAGGCAGTGCGCTGGG	GGATCCGGTTGCGTTGATCGCGCTC
alg-2	CGCGGTACCCCACTGACTTCATGCACATGTCTACTTAG	CGCGGATCCTTCTGAAAACATTTTATTTGATAATGAGAAGC
cbp-1	AAAGGTACCCGAACATGACCGATTGTAGA	AAAAGATCTATACATTTTTCTACTAATTG
cgh-1	AAAGCTAGCCGCCTCCTCATCGTCAGATGTC	GGAGGATCCTTTCCGATGTCGTAGTAGGTTTGATTTCCTG
daf-3	AAAGGTACCACATTAGAGTGGAAATAGTA	CCCGGATCCTTATGTGTACTCTGACTATT
daf-16B	GGAGCTAGCCTGAACTTGTCGCTCGAAAGGATG	GGAGGATCCAACGTCTTCGGGAATTTCAGCCAAAGACGACG
din-1S	PCR from pPD118.15 din-1S:GFP (strain AA790) with forward primer GCGGAATTCGGCTGAAATCACTCACAACGATGGATACG and reverse primer GGGTATCTCGAGAAGCATTGAACACC and cloned into pPD95.75 with HindIII and XhoI. Then subcloned as a XmaI - XhoI fragment.	
dre-1	GCAGCTAGCGCCAGTTCCCATGCTAATCTTTAGAAATGC	AAAGGATCCCTCCTGGCCAACCAGAGACGATGTCGG
let-70/atg-7	AAAGCTAGCCCTTGTTGCAGCCTCGTGTTCATCTCG	AAAGGATCCGTTGGTGTTCTGATTTGCTGATTGACCC
lin-28	AAAGCTAGCCTTCCAGGTTACGGTAGTTTTAAAGGCAC	AAAAGATCTTGTTCTAATTAGAACTATGTGTTAACTAGAGAATACAACC
lin-41	GCGTCTAGACAAAGAAAAATATAAGAAATCATCTGG	AAAGGATCCTTCACTTTTTCCAAGTCTG
lin-42A	TTGGTCGACATTCCCCCTGAAAAGCATCT	AAAGGATCCTTTAGGGTGGTAGGCTT
nhl-2	AGATCTAGACACCTTATCCCAGTCATCGTC	AAAGGATCCCTTCACGGAATGAACGTTCG
kin-20	GGAGCTAGCGAGTGAAGAGAAATAGAGAAAAGTGCAAATGTC	AGAGGATCCACACCTCTCTGCACAATCGGGAC

Oligos to subclone promoters into pGL3 Luc vector.

Gene	Forward primer	Reverse primer
ain-1	Same fragment as used for pGL3 Luc vector.	
alg-2	Same fragment as used for pGL3 Luc vector, but digested with BamHI and Pst I (from the sequence).	
lin-42A	TTGGTCGACATTCCCCCTGAAAAGCATCT	TCTGGATCCAAGATCGTACAAGTAGCTTAA

Oligos to subclone promoters into pPD95.75 vector.
